# Autonomic regulation therapy to enhance myocardial function in heart failure patients: the ANTHEM‐HFpEF study

**DOI:** 10.1002/ehf2.12241

**Published:** 2017-12-28

**Authors:** Lorenzo A. DiCarlo, Imad Libbus, H. Uday Kumar, Sanjay Mittal, Rajendra K. Premchand, Badri Amurthur, Bruce H. KenKnight, Jeffrey L. Ardell, Inder S. Anand

**Affiliations:** ^1^ LivaNova PLC London UK; ^2^ Yashoda Hospital Secunderabad India; ^3^ Medanta—The Medicity New Delhi India; ^4^ Krishna Institute of Medical Science Secunderabad India; ^5^ University of California—Los Angeles Los Angeles CA USA; ^6^ Minneapolis VA Health Care System, University of Minnesota Cardiology 111 C, One Veterans Drive Minneapolis MN 55417 USA

**Keywords:** Autonomic balance, Heart failure, Preserved ejection fraction, Neuromodulation, Autonomic regulation therapy, Vagus nerve stimulation

## Abstract

**Background:**

Approximately half of the patients presenting with new‐onset heart failure (HF) have HF with preserved left ventricular ejection fraction (HFpEF) and HF with mid‐range left ventricular ejection fraction (HFmrEF). These patients have neurohormonal activation like that of HF with reduced ejection fraction; however, beta‐blockers and angiotensin‐converting enzyme inhibitors have not been shown to improve their outcomes, and current treatment for these patients is symptom based and empiric. Sympathoinhibition using parasympathetic stimulation has been shown to improve central and peripheral aspects of the cardiac nervous system, reflex control, induce myocyte cardioprotection, and can lead to regression of left ventricular hypertrophy. Beneficial effects of autonomic regulation therapy (ART) using vagus nerve stimulation (VNS) have also been observed in several animal models of HFpEF, suggesting a potential role for ART in patients with this disease.

**Methods:**

The Autonomic Neural Regulation Therapy to Enhance Myocardial Function in Patients with Heart Failure and Preserved Ejection Fraction (ANTHEM‐HFpEF) study is designed to evaluate the feasibility, tolerability, and safety of ART using right cervical VNS in patients with chronic, stable HFpEF and HFmrEF. Patients with symptomatic HF and HFpEF or HFmrEF fulfilling the enrolment criteria will receive chronic ART with a subcutaneous VNS system attached to the right cervical vagus nerve. Safety parameters will be continuously monitored, and cardiac function and HF symptoms will be assessed every 3 months during a post‐titration follow‐up period of at least 12 months.

**Conclusions:**

The ANTHEM‐HFpEF study is likely to provide valuable information intended to expand our understanding of the potential role of ART in patients with chronic symptomatic HFpEF and HFmrEF.

## Introduction

Approximately 50% of patients with heart failure (HF) have preserved and mid‐range left ventricular ejection fraction (LVEF; ≥40%) with or without features of diastolic dysfunction.^1^ Hallmarks of diastolic dysfunction include left atrial enlargement, elevated *E*/*e*′ ratio [the peak mitral velocity of early filling (*E*) divided by early diastolic mitral annular velocity (*e*′)], left ventricular hypertrophy, and elevated circulating natriuretic peptides.^1^, ^2^, ^3^ The pathological changes in the left ventricle (LV) include myocyte hypertrophy, apoptosis, necrosis, and excessive interstitial collagen deposition in the myocardium.^4^ As in patients with HF with reduced ejection fraction (HFrEF), patients with HF with preserved LVEF (HFpEF; LVEF ≥ 50%) and HF with mid‐range LVEF [HFmrEF; ejection fraction (EF) = 40–49%] also have significant neurohormonal activation with poor outcomes.^5^, ^6^, ^7^

While there have been significant therapeutic advances for patients with HFrEF through the use of neurohormonal antagonists and device‐based therapies such as cardiac resynchronization, definitive treatment interventions for patients with HFpEF and HFmrEF have been not been identified.^8^ In particular, beta‐blockers and angiotensin‐converting enzyme inhibitors have not been shown to improve outcomes. Only two agents, angiotensin receptor blockers (ARBs) and aldosterone receptor antagonists (ARAs), have been shown to reduce hospitalizations in selected HFpEF patients.^9^, ^10^ Recent guidelines list both these agents as Class IIb therapies, and current treatment of HFpEF and HFmrEF remains symptom based and empirical.^11^ In addition to ARBs and ARAs, pharmacological management typically consists of diuretics for the amelioration of symptomatic volume overload, rate control for patients with atrial fibrillation, and management of co‐morbidities such as hypertension.^12^, ^13^

There is considerable rationale for testing the use of autonomic modulation with vagus nerve stimulation (VNS) in patients with HFpEF and HFmrEF. Autonomic dysregulation is an important component of HFpEF and HFmrEF, and patients with HFpEF and HFmrEF show a similar pattern of neurohormonal activation to those with HFrEF.^8^ Vagus nerve stimulation increases parasympathetic activity, reduces sympathetic tone, and stabilizes the neural network in the autonomic nervous system regulating cardiovascular function.^14^ Increased parasympathetic activity results in increased muscarinic receptor activation and decreases excess adrenergic receptor activation.^15^ Muscarinic receptor activation at the level of the cardiac myocyte reduces oxidative stress, increases contractile function, improves calcium signalling function in the heart, and normalizes gene expression.^15^, ^16^ At the same time, cholinergic trans‐differentiation of sympathetic neurons takes place, providing a protective role against sympathetically mediated pathogenesis.^17^ In canine and guinea pig models of hypertension‐mediated HF, chronic VNS was shown to mitigate hypertrophy and reverse multiple adverse changes in autonomic control of the heart, including myocyte size and LV mass, findings that are particularly relevant to HFpEF.^18^, ^19^

Although VNS has never been clinically evaluated in HFpEF and HFmrEF patients, several clinical studies have been conducted in HFrEF patients. The Neural Cardiac Therapy for Heart Failure (NECTAR‐HF) study was a randomized controlled study that evaluated the effect of right cervical VNS in 96 patients with HFrEF.^20^ This study failed to meet its primary endpoint, 6‐month improvement in LV end systolic diameter, although quality of life and New York Heart Association (NYHA) class were both significantly improved. As the investigators themselves note, the failure of the NECTAR‐HF study was likely due to the high frequency of stimulation of the vagus nerve (20 Hz), which elicited patient intolerance and prevented titration to a therapeutic dose. The Increase of Vagal Tone in Heart Failure (INOVATE‐HF) study, a pivotal trial in 707 patients, was stopped early for futility. The study failed to meet its primary endpoint, reduction in death or HF events, despite significant improvements in several secondary endpoints (NYHA class, quality of life, and 6 min walk distance).^21^ Post hoc analysis of the INOVATE‐HF study demonstrated that adequate stimulation levels were not reached across all patients and that poor response to VNS was seen in 30% of patients with cardiac resynchronization therapy devices.

Autonomic Neural Regulation Therapy to Enhance Myocardial Function in Heart Failure (ANTHEM‐HF) studied 60 patients with HFrEF and demonstrated that VNS is safe, is feasible, and results in improvements in cardiac function and HF symptoms.^22^, ^23^ In that study, stimulation was delivered at a moderate intensity (2.0 ± 0.6 mA) and a frequency of 5–10 Hz (near the natural frequency of discharge of vagal fibres during physiological reflex activation^24^, ^25^). The device used in the ANTHEM‐HF study (VITARIA, LivaNova PLC, London, UK) received Conformité Europeénne Mark approval in 2015 for the treatment of HFrEF.^26^

The Autonomic Neural Regulation Therapy to Enhance Myocardial Function in Patients with Heart Failure and Preserved Ejection Fraction (ANTHEM‐HFpEF) study is designed to evaluate the feasibility, tolerability, and safety of autonomic regulation therapy (ART) using right cervical VNS in patients with chronic symptomatic HFpEF and HFmrEF.

## Methods

### Study design

The ANTHEM‐HFpEF is an open‐label, multicentre, single‐arm study. It is anticipated that approximately eight study sites will enrol approximately 50 study subjects. Each study subject will participate in the study for at least 15 months.

### Device implantation and vagus nerve stimulation titration

The VNS Therapy System (LivaNova PLC) will be used to deliver ART for this study. The functionally equivalent VITARIA™ system received Conformité Europeénne Mark approval in 2015 for the treatment of HFrEF patients who have symptomatic moderate to severe HF (NYHA Class II/III) with LV dysfunction (LVEF ≤ 40%), despite stable, optimal HF drug therapy. The system has been described in detail previously.^27^

The ART system will be implanted subcutaneously, following the approved implantation procedure for patients with HF. The lead, including bipolar electrodes (anode cephalad to cathode), will be placed on the right cervical vagus nerve through a transverse incision in the neck halfway between the clavicle and the mastoid process. The lead body will be tunnelled subcutaneously from the neck incision site to the pulse generator in an ipsilateral, subclavicular chest pocket. After a 2‐week post‐implantation recovery period and a 10‐week stimulation titration period, continuous cyclic stimulation will be delivered for at least 12 months.

### Study objectives

The main objectives of the ANTHEM‐HFpEF are to evaluate the feasibility, tolerability, and safety of ART via VNS for the treatment of patients with HFpEF and HFmrEF. The study will also seek to identify signals of clinical improvement. Feasibility is defined as the percentage of study subjects who are successfully implanted with the lead and pulse generator, based upon implant attempt. Tolerability is defined as the percentage of study subjects who continue therapy throughout the follow‐up period that begins after post‐implantation titration of VNS is complete. Safety is defined as the incidence of procedure‐related and device‐related adverse events.

The pilot study will also seek to identify signals of cardiovascular and functional improvement in patients with HFpEF and HFmrEF. The following efficacy measurements will be made at baseline and at each follow‐up visit: echocardiographic assessment of cardiac structure and function including left atrial volume index, LV mass, and Doppler indices of LV diastolic function; measurements of functional status such as NYHA class, quality of life using the Minnesota Living with Heart Failure Questionnaire, 6 min walk distance, mean heart rate, and heart rate variability during 24 h Holter electrocardiography; and plasma biomarkers including N‐terminal pro‐BNP and high‐sensitivity C‐reactive protein.

### Study population

**Figure 1:**
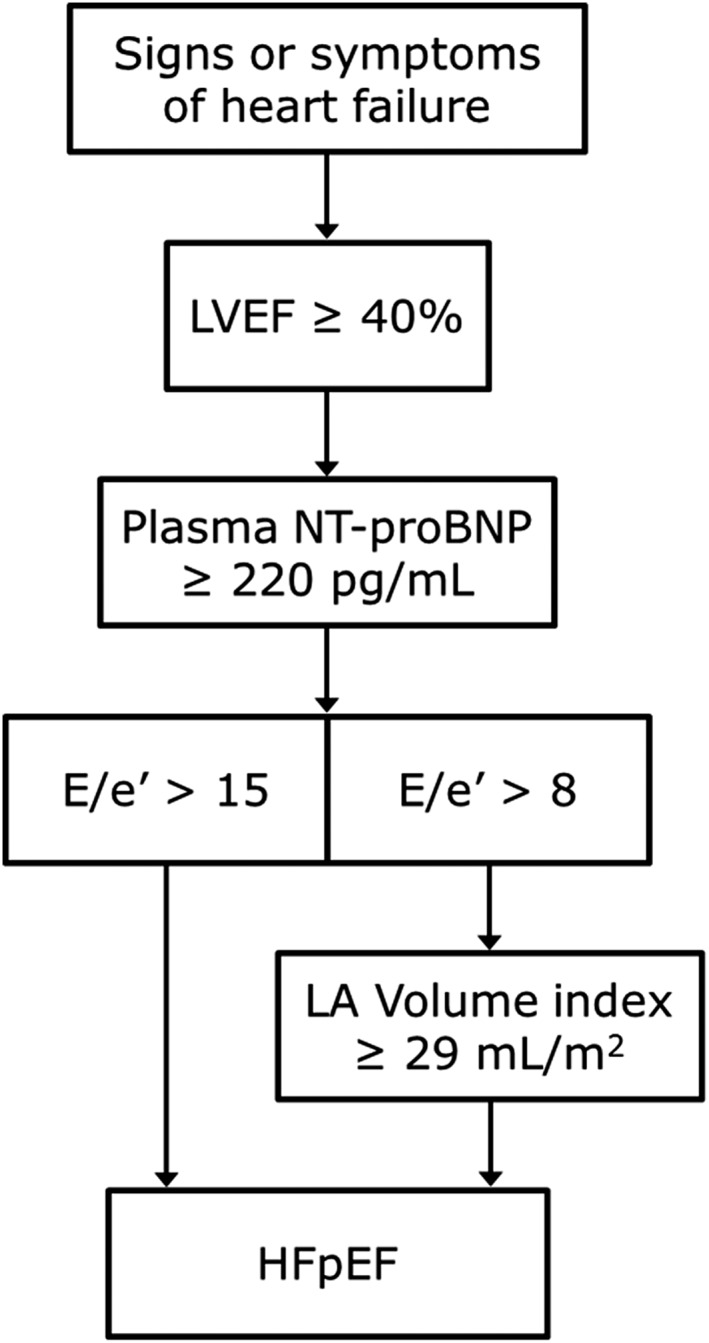
Key inclusion criteria for determining HFpEF patients eligible for enrolment in the Autonomic Neural Regulation Therapy to Enhance Myocardial Function in Patients with HFpEF study. HFpEF, heart failure with preserved ejection fraction; LVEF, left ventricular ejection fraction; NT, N‐terminal.

Male and female patients 18 years of age or older, with NYHA Class II or III HF symptoms, on stable guideline‐directed pharmacologic therapy including a loop diuretic for at least 1 month, with controlled systolic blood pressure (<140 mmHg) or systolic blood pressure between 140 and 160 mmHg, and receiving three or more blood pressure medications will be eligible for enrolment. Other key inclusion criteria include LVEF ≥ 40%, plasma N‐terminal pro‐BNP ≥ 220 pg/mL, ratio of mitral inflow velocity to early diastolic velocity of the mitral annulus (*E*/*e*′) > 15, or *E*/*e*′ > 8 and left atrial enlargement (left atrial volume index ≥ 29 mL/m^2^) (*Figure* 1). Subjects must be physically capable and willing to perform repeated 6 min walk tests and achieve a baseline distance of between 150 and 425 m that is limited by symptoms due to HF.

Exclusion criteria include HF due to congenital heart disease, hypertrophic or restrictive cardiomyopathy, a recent HF hospitalization or intravenous HF therapy in the past 30 days, therapeutic cardiovascular intervention or surgery within the past 2 months or planned within the next 6 months, participation in an investigational drug or device trial currently or in the past 3 months, or suffering from a medical or surgical condition that could reduce life expectancy or present an unacceptable risk from ART system implantation or VNS.

### Study treatment

Subjects who fulfil all of the inclusion and exclusion criteria and provide written informed consent will receive thoracic subcutaneous ART therapy system implantation for right cervical VNS. After a 2‐week post‐implant recovery period, the device will be activated and VNS will be initiated at a current output of 0.25 mA, pulse frequency of 5 Hz, pulse width of 130 μs, and a duty cycle of 17.5% (14 s on/66 s off). The titration period will have a duration of approximately 10 weeks. During the titration period, stimulation parameters will be adjusted during weekly titration sessions in a prescribed stepwise fashion under continuous electrocardiography monitoring and recording to a maximum current output of 3.0 mA, pulse frequency of 5 Hz, pulse width of 250 μs, and a duty cycle of 17.5% (14 s on/66 s off), limited by VNS‐related side effects such as activation of the expiratory reflex (mild cough), intolerable stimulation sensation, or acute heart rate reduction (>4 b.p.m.) during VNS active phase. Once the titration period is complete, VNS therapy will continue for at least 12 months.

Subjects will be instructed regarding potential adverse device‐related effects and will be provided a magnet to inhibit pulse generator stimulation in the event of any intolerable device‐related effects. Should subjects perform this intervention, they will be instructed to contact the site investigator, who will assess whether an adverse device‐related event has occurred and whether the programmed settings should be changed.

### Study analysis

All echocardiographic recordings and blood samples will be sent to a designated core laboratory facility for analysis. Data will be batched for analysis, and analysis will incorporate blinding to the study subject and the temporal order of the data being analysed. All adverse events will be reviewed by an independent clinical events committee that will adjudicate adverse event severity and whether or not an adverse event is device related. An independent data and safety monitoring committee will oversee the study, and the study will be analysed using the following statistical analysis plan.

Because the focus of the study is on feasibility and safety, the sample size for this study was not statistically derived. Data from all investigational sites will be pooled for analysis. Standard statistical methods will be employed to analyse all data. Continuous variables will be summarized using the number of observations, mean, median, standard deviation, and minimum and maximum values. Variables that are not normally distributed will be log transformed. Categorical variables will be summarized using the number of observations and percentages. Statistical significance testing will be performed at the 0.05 level. All tests of hypotheses will be two‐tailed.

## Study risks and mitigations

The most commonly reported side effects of VNS include mild cough, voice alteration, paraesthesia, nausea, dyspepsia, and dyspnoea. Vagus nerve stimulation is also known to affect the sinoatrial and atrioventricular nodes. The protocol requires stimulation titration below the threshold of significant cardiac (heart rate and atrioventricular conduction) effects; however, there is a risk that stimulation could cause mild acute increase or decrease in heart rate, depending on the VNS parameters that are utilized.^28^

The study is designed to mitigate each of these risks. Subjects will be monitored for symptomatic tolerability and any significant increase or decrease in heart rate while the intensity of VNS is being titrated in a clinical setting. Subjects will be observed weekly during therapy titration, and cardiac rhythm will be assessed. If atrial fibrillation develops and is considered to be related to VNS, the clinical investigators may temporarily or permanently discontinue VNS. The subjects will also be observed closely during the first 12 weeks of the study, and if worsening HF is observed, investigators may consider discontinuing VNS.

## Discussion

Approximately half of the patients presenting with new‐onset HF have HFrEF, and half have HFpEF and HFmrEF. The LV of patients with HFrEF is generally enlarged and may appear thin walled on echocardiography with an EF ≤ 40%. In contrast, patients with HFpEF and HFmrEF typically have LVs that are generally normal or thick walled, with LVEF ≥ 40%, and LV volume that is reduced. The 2016 updates of guidelines for the treatment of HF introduced a new category of HF with mid‐range LVEF to describe patients with HF and LVEF ranging from 40% to 49% (HFmrEF), reserving HFpEF to describe patients with HF and LVEF ≥ 50%. The ANTHEM‐HFpEF study was designed and launched before these guideline revisions were introduced and includes both HFpEF and HFmrEF in the study population. The characteristics and outcomes of patients with HFmrEF currently appear to be similar to those with HFpEF, and there are currently limited evidence‐based treatments for patients with an LVEF of 40% or greater.^11^, ^12^, ^13^

Despite differences in the hearts of patients with HFrEF, HFpEF, and HFmrEF, the signs, symptoms, and other clinical manifestations are very similar in all three conditions. The pathophysiological mechanisms of salt and water retention are also very similar. In both conditions, there is an autonomic imbalance with increase in sympathetic and renin–angiotensin–aldosterone system activity and withdrawal of parasympathetic efferent activity.^29^ However, unlike the dramatic improvement in outcomes seen with the use of blockers of the sympathetic and renin–angiotensin–aldosterone system in patients with HFrEF, beta‐blockers and angiotensin‐converting enzyme inhibitors have not been demonstrated in patients with HFpEF or HFmrEF, and ARBs and ARAs have only a modest effect on HF hospitalization. Whether enhancing parasympathetic tone by VNS may be beneficial in patients with HFpEF and HFmrEF has not been examined. However, existing pre‐clinical and clinical evidence is available, demonstrating that sympathoinhibition using parasympathetic interventions leads to improvements in central and peripheral neural network functions, baroreceptor reflexes, myocyte energetics, and regression of LV hypertrophy.^15^, ^17^, ^30^, ^31^

Studies in a guinea pig model of pressure‐overload LV hypertrophy have shown improvement in echocardiographic features of LVH and LV filling, suggesting a potential role of ART in HFpEF and HFmrEF.^18^ In a canine model of HFpEF using bilateral non‐restrictive renal wrapping, which induced increased blood pressure and increased LV mass, ART induced a significant regression of LV mass and enhanced inotropic and lusitropic response to direct stellate stimulation, suggesting that ART improved cardiovascular autonomic control.^19^ Early clinical studies of ART have shown promising safety and efficacy trends when VNS is properly titrated to a defined target in appropriately selected patients with HFrEF,^22^, ^23^, ^32^ and there are two ART systems currently approved for clinical use for HFrEF in Europe.^26^, ^33^

Based upon these considerations, the ANTHEM‐HFpEF study has been designed to examine the feasibility, tolerability, safety, and potential benefits of ART via VNS using the LivaNova VNS Therapy System in patients with EF ≥ 40%.

## Summary

By investigating the feasibility, tolerability, and potential benefits of ART in patients with HFpEF and HFmrEF and complementing the previously published ANTHEM‐HF study in patients with HFrEF, the ANTHEM‐HFpEF study will serve to expand our understanding of the role of ART in patients with chronic symptomatic HF.

## Conflict of interest

Drs Ardell, and Anand are medical and scientific advisors to LivaNova. Dr DiCarlo, Dr Libbus, Mr Amurthur, and Dr KenKnight are employees of LivaNova. Drs Kumar, Mittal, and Jain are investigators in the ANTHEM‐HFpEF study.

## Funding

This work was supported by LivaNova.
